# Intermediate-risk prostate cancer patients treated with androgen deprivation therapy and a hypofractionated radiation regimen with or without image guided radiotherapy

**DOI:** 10.1186/1748-717X-8-137

**Published:** 2013-06-07

**Authors:** Maurizio Valeriani, Stefano Bracci, Mattia Falchetto Osti, Teresa Falco, Linda Agolli, Vitaliana De Sanctis, Riccardo Maurizi Enrici

**Affiliations:** 1 Department of Radiation Oncology, La Sapienza University, Sant’Andrea Hospital, Via di Grottarossa 1035-1039, Rome, 00189, Italy

**Keywords:** Intermediate-risk prostate cancer, Hypofractionated radiotherapy, IGRT, 3D-CRT

## Abstract

**Background:**

To evaluate the efficacy of hypofractionated radiotherapy (HyRT) with or without image guided radiotherapy (IGRT) in intermediate risk prostate cancer.

**Methods:**

105 patients were treated with HyRT, 43,8 Gy and 54,75 Gy were delivered to the seminal vescicles and to the prostate, respectively; 3,65 Gy/fraction three times weekly. All patients underwent 9 months hormonal therapy. Patient position was verified with daily kV cone beam CT in 69 patients (IGRT group). Acute and late toxicities were evaluated according to RTOG scale. Biochemical relapse was defined using PSA nadir + 2 ng/mL. The data were prospectively collected and retrospectively analyzed to evaluate the efficacy of IGRT.

**Results:**

After a median follow-up of 31 months the actuarial 3-year bNED was 93,7%. During RT, 10.5% and 7.6% of patients developed ≥Grade 2 rectal and urinary toxicities, respectively. The cumulative incidence of ≥Grade 2 late rectal and urinary toxicities at 3 years were 6,9%, and 10,8%, respectively. The incidence of ≥Grade 2 late rectal toxicities was significant reduced in the IGRT group (1,6% vs. 14,5%, p=0,021). Two patients developed Grade 3 urethral obstruction and one patient developed grade 3 rectal bleeding.

**Conclusions:**

HyRT represents a well-tolerated treatment able to achieve a high bNED. The use of daily IGRT is beneficial for reducing the incidence of late toxicities.

## Background

External Beam Radiotherapy (RT) is one of the therapeutic options for treating prostate cancer. The use of high dose RT with a conventional fractionation delivered conformally to spare as much normal tissue as possible, results in a significant biochemical control with acceptable toxicities [1].

In the past decade hypofractionated radiotherapy (HyRT) has been proposed as an alternative to conventional fractionation. Unlike most tumors, the estimated α/β ratio of prostate cancer is about 1.5 Gy, showing lower value compared to the α/β of surrounding tissues (e.g. rectum) [2]. Prostate cancer, because of its intrinsic slow proliferation, would be more sensitive to fractionations than nearby late-responding tissues. According to the Linear-Quadratic Model (LQM), the use of fewer and larger fractions of radiation instead of the conventional daily fractions of 2 Gy, would lead to an expected therapeutic advantage [3]. Moreover, HyRT is convenient for patients since the overall treatment time is reduced of several weeks depending on the higher dose per fraction, as well as for costs reduction of the health care system.

Randomized phase 3 trials comparing HyRT vs. conventional RT were carried out in late 1990s, but results are not conclusive because total doses used both in the control arm (64–66 Gy) and in the hypofractionated arm (biological equivalent dose of 62–67 Gy) are considered to be below the current standard [4,5]. The recent report on clinical outcome of the RTOG 9406 trial, concluded that dose-escalated 3D-CRT yields favorable outcomes for localized prostate cancer [6]. In the past decade differents fractionations have been explored in several randomized and non-randomized trials with good biochemical control and with acceptable acute and late toxicity profile [7-9]. Furthermore, the progress in radiotherapy techniques capable to spare healthy tissues allowing a dose-escalation such as intensity modulated RT (IMRT), stereotactic body RT (SBRT) or the use of image-guided RT (IGRT) would probably lead to a better clinical outcome [10-15].

To assess this issue, in 2007 we started a study using a novel HyRT regimen with or without IGRT for intermediate risk prostate carcinoma. A preliminary report regarding acute toxicity on 62 patients enrolled was previously published [16]. In the present paper, the authors assessed acute and late toxicity rates and provide survivals data in a cohort of 105 intermediate risk patients. Subgroups’ analysis was also performed to evaluate any difference related to the use or not of daily IGRT in terms of toxicity or biochemical no evidence of disease (bNED).

## Methods

### Patients’ characteristics

Between March 2007 and June 2011, 105 consecutive patients affected by intermediate risk prostate cancer were prospectively enrolled. Under an IRB-approved protocol the data were prospectively collected and retrospectively analyzed to evaluate the efficacy of IGRT in terms of toxicities and bNED. Written consent was obtained from all patient. All patients had histologically confirmed diagnosis and Gleason Score definition using transrectal ultrasound (TRUS) guided biopsies. The pre-treatment evaluation included patients’ complete history, physical examination with digital rectal examination (DRE), blood tests including PSA level, total body computed tomography (CT) scan with iodine-based contrast and 99mTc bone scan. TRUS was employed for local staging in the early group of patients included (n=36, non-IGRT group), while multiparametric magnetic resonance imaging (MRI) of the pelvis including diffusion-weighted imaging (DWI) and dynamic contrast-enhanced study (DCE-MRI) was obtained in the following group of patients (n=69, IGRT group).

According to the National Comprehensive Cancer Network (NCCN) guidelines, intermediate risk group includes patients with any clinical T2b–T2c prostate cancer, Gleason Score equal to 7 or pre-treatment PSA value ranging from 10 to 20 ng/mL [17].

### Simulation and treatment

Prior to simulation, patients were educated to empty the rectum and fill the bladder. All patients were immobilized in the supine position with feet fixation. A pre-treatment CT planning with 2.5 mm slices from the anal verge to the L5–S1 interface was performed. Planning CT images were fused with the multiparametric MRI to better delineate the Clinical Target Volume (CTV) in the IGRT group (n=69). The CTV1 included the prostate plus seminal vesicles (SSVV) and the CTV2 the prostate alone. Planning Target Volumes (PTV1 and PTV2, respectively) were generated adding a 8 mm margin in all directions except in the posterior direction where a 6 mm expansion was adopted in the non-IGRT group (n=36). A 5 mm expansion in all direction was used for the IGRT group. Indeed, the use of daily CBCT permit to reduce set-up errors as well the internal margin due to the interfraction motion of the prostate. The margin of 5 mm has been chosen to encompass the intrafraction movement also taking into account the deformation of the target as well as of the organ at risk (i.e. bladder and rectum) during the treatment.

The whole rectum from the anus to the sigmoid flexure, bladder, femoral heads and penile bulb were contoured as organs at risk.

A 3D conformal radiotherapy (3D-CRT) plan on the Eclipse planning system (Varian, Palo Alto, CA) was performed using 5 coplanar fields. Treatment was delivered by a linear accelerator using 15 MV photons. Thus the PTV1 received 43.8 Gy in 12 fractions (3.65 Gy per fraction) and the PTV2 received 54.75 Gy in 15 fractions, three times a week. According to the LQM this RT regimen is biologically equivalent to 80.5 Gy in 2 Gy fractions assuming a α/β ratio of 1.5 Gy. This regimen is also equivalent to 72.8 Gy in 2 Gy fractions assuming a α/β ratio of 3 Gy for late responding tissue. The overall treatment time has been chosen based on studies on acute mucosal toxicity in head and neck cancers showing that a BED10 over 60 Gy may be associated with a higher acute toxicity rate [2]. Thus, with the aim to maintain the acute toxicity rate and the consequential late damage low, we decided not to shorten below 5 weeks the overall treatment time. [18]. Dose–volume constraints were as follows: V45<35% and V52<25% for the rectum; V40<50% for the bladder.

Patient position was verified using electronic portal imaging device (EPID) in the non-IGRT group, while the IGRT group underwent daily kV cone-beam CT (CBCT).

Neoadjuvant, concomitant and adjuvant hormonal therapy (HT) was started 3 months before RT. The HT consisted in anti-androgen or LHRH-analogue according to the treating physician’s preference and was administered to all patients.

### Toxicity and follow-up

Follow up was performed every 3 months for the first year and every 6 months afterwards. Toxicities were prospectively assessed according to the Radiation Therapy Oncology Group (RTOG) scale for acute and late adverse effects at each follow-up [19]. In general, the occurrence of symptoms not requiring medications or an increased in magnitude of baseline symptoms were considered as Grade 1 toxicities. Grade 2 toxicities were considered as the occurrence of symptoms requiring new medications (e.g. antidiarrheal drugs) or increase of previously prescribed medication or symptoms requiring a single surgical intervention (i.e. single laser coagulation). Grade 3 toxicities were defined as the occurrence of symptoms requiring surgery (i.e. TURP or permanent catheter or bleeding requiring ≥2 laser coagulation). Late toxicities were defined as occurring after 90 days from the completion of treatment. Erectile dysfunction was also evaluated using the following question from the Sexual Adjustment Questionnaire: “When sexually excited, are you able to get an erection?” at baseline, 3 months after RT and 1 year after HT. The five levels of response were: always, almost always, sometimes, almost never, never, not applicable/not answer [20].

### Statistical analysis

The biochemical failure was defined as the PSA nadir + 2 ng/mL according to the Phoenix criteria [21]. Overall survival (OS), disease specific survival (DSS), bNED and the cumulative incidence of ≥ Grade 2 late toxicity were calculated to the event using the Kaplan–Meier method. In the subgroups’ analysis acute toxicities between the IGRT and non-IGRT series were compared using the chi-square test. Differences in bNED as well the cumulative incidence of ≥ Grade2 late toxicities between the two groups were evaluated with log-rank test. Statistical analyses were performed using SPSS statistical software package version 13.0 (SPSS, Inc., Chicago, IL). A *p*-value of less than 0.05 was considered to be statistically significant.

## Results

### Patients’ characteristics and survivals

Median age at diagnosis was 74 years (range, 55–88 years). Thirty-three patients had Gleason 6 (3+3) and 7 patients presented with T1c clinical stage. Median PSA level at diagnosis was 10 ng/mL (range, 2–19,9). Patients’ characteristics are summarized in Table 1.

**Table 1:** Patients’ characteristics

**Characteristics**	**Non IGRT**		**IGRT**		**Total**		p value*
	**(n=36)**		**(n=69)**		**(n=105)**		
	**n.**	**%**	**n.**	**%**	**n.**	**%**	
**Age**							
*<75*	15	41,7	40	58	55	52,4	0,112
*≥75*	21	58,3	29	42	50	47,6	
**PSA at the diagnosis (ng/mL)**							
*0.1- 10*	22	61,1	33	47,8	55	52,4	0,196
*10.1 -19.9*	14	38,9	36	52,2	50	47,6	
**Gleason Score**							
*3+3*	8	22,2	25	36,2	33	31,4	0,340
*3+4*	19	52,8	30	43,5	49	46,7	
*4+3*	9	25	14	20,3	23	21,9	
**Clinical Stage**							
*T1c*	6	16,7	1	1,4	7	6,7	0.011
*T2a*	13	36,1	20	29	33	31,4	
*T2b*	8	22,2	28	40,6	36	34,3	
*T2c*	9	25	20	29	29	27,6	
**Hormonal Therapy**							
*Anti-androgen*	23	63,9	53	49,9	76	72,4	0.160
*GnRH agonist*	13	36,1	16	19,1	29	27,6	

*Chi-square test.

**Figure 1:**
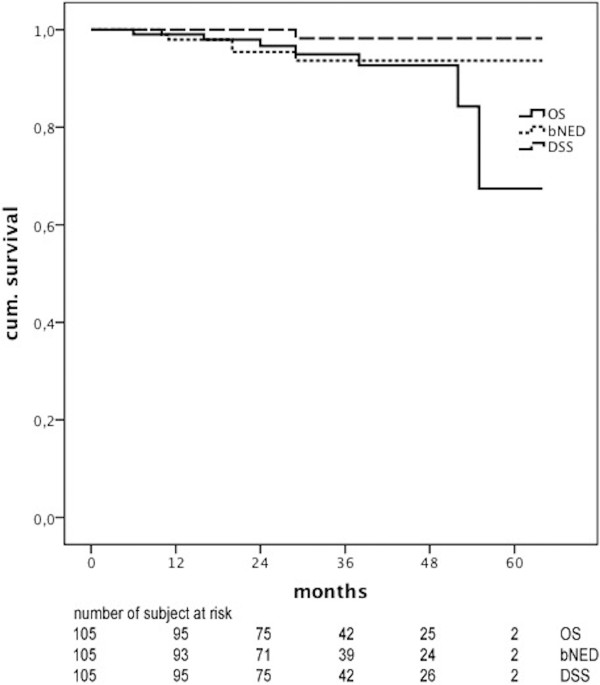
Overall survival, disease-specific survival and biochemical no evidence of disease.

After a median follow-up of 31 months (range, 6–64 months), the actuarial 3-year BFS was 93,7% (Figure 1). The median follow-up was 50 months (range, 7–64 months) and 25 months (range, 6–43 months) for non-IGRT and IGRT patients, respectively. Five patients developed biochemical failure: 3 patients were found to have metastasis to loco-regional lymph nodes and 2 patients had bone metastasis. Subsequently, all these patients (n=5) were submitted to total Androgen Deprivation Therapy (ADT). Patients with regional lymph nodes received salvage radiation therapy based on 18FCholine-PET/TC. Patients with bone metastasis received palliative radiation treatment. Then, PSA levels normalization was obtained in 3 patients (node metastasis: 2 patients; bone metastasis: 1 patient). One patient with lymph-node metastasis did not respond to ADT plus RT and underwent taxane-based chemotherapy that is still ongoing. The other patient with bone metastasis died because of systemic disease progression. Six patients died from intecurrent disease without evidence of prostate cancer. The 3-year OS was 95% and the 3-years DSS 98,2% (Figure 1). No differences were found in BFS between the two groups of patients (91,3% vs. 94,3%; p=0,413).

### Acute toxicities

During RT, 15.2% of the patients developed Grade 1 GI toxicity and 10.5% developed ≥ Grade 2 GI toxicity. At 3 months after RT, 4 patients had Grade 1 GI toxicities and only 1 patient had a Grade 2 proctitis. Acute GU toxicities during RT were recorded as follows: 47.6% experienced Grade 1 toxicities and 7.6% experienced ≥ Grade 2 toxicities. At 3 months after RT, 31.4% had Grade 1 GU toxicities and 2.9% had ≥ Grade 2 GU toxicities. None had grade 3–4 acute toxicities (Table 2).

**Table 2:** Comparison of acute rectal and urinary toxicities in the no IGRT group vs. IGRT group

**Acute toxicity**	**Rectal**					**Urinary**				
	**no IGRT**		**IGRT**		p value*	**no IGRT**		**IGRT**		p value*
	**n.**	**%**	**n.**	**%**		**n.**	**%**	**n.**	**%**	
**during RT**										
G1	8	22.2	8	11.6	0.105	18	50	32	46.4	0.724
≥G2	2	5.6	9	13	0.234	3	8.3	5	7.2	0.842
**3 months FU**										
G1	2	5.6	2	2.9	0.5	13	36.1	20	29	0.455
≥G2	0	0	1	1.4	0.468	1	2.8	2	2.9	0.972

* Chi-square test.

### Late toxicities

**Figure 2:**
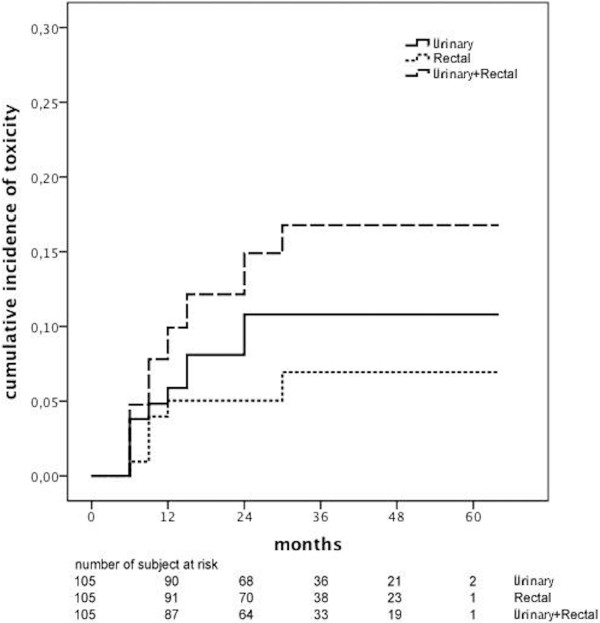
Cumulative incidence of rectal, urinary and rectal + urinary ≥ Grade 2 late toxicities.

**Figure 3:**
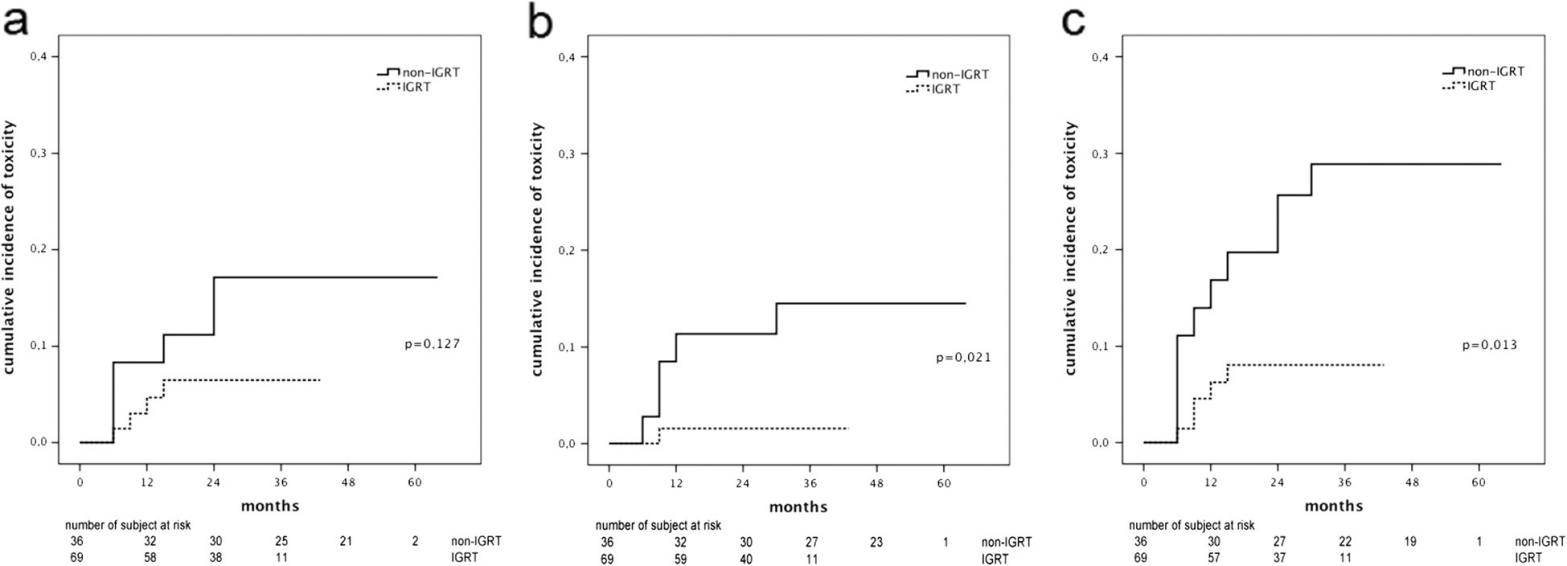
Comparison of (a) urinary, (b) rectal and (c) rectal + urinary ≥ Grade 2 late toxicities in the non-IGRT and IGRT patients.

A total of 10 patients (9,5%) developed ≥ Grade 2 GU toxicities and 6 patients (5,7%) developed ≥ Grade 2 GI toxicities. The prevalence of GI and GU toxicities according to RTOG scale are shown in Table 3. The cumulative incidence of ≥ Grade 2 GI toxicities at 3 years was 6,9%, while the cumulative incidence of ≥ Grade 2 GU toxicities at 3 years was 10,8% (Figure 2). Two patients developed Grade 3 urethral obstruction at 6 months from RT completion (1 permanent catheter and 1 TURP). One patient developed grade 3 rectal bleeding at 6 months that required repeated argon plasma coagulation. None developed Grade 4 toxicity. The cumulative incidence of ≥ Grade 2 GI toxicities was significant reduced in the IGRT group (1,6% vs. 14,5%, p=0,021) as well as the cumulative incidence of both GI and GU ≥ Grade 2 toxicities (28,9% vs. 8,1%, p=0,013) (Figure 3b and c). On the other hand, the cumulative incidence of ≥ Grade 2 GU toxicities resulted not statistically different in the two groups (6,5% vs. 17,1%, p=0,127) (Figure 3a).

**Table 3:** Prevalence of toxicities according to RTOG scale

**Follow-up (months)**		**During RT**	**3**	**6**	**9**	**12**	**18**	**24**
**Toxicity**	**Grade**	**%**	**%**	**%**	**%**	**%**	**%**	**%**
**Bowel frequency**	0	87	99	98	98	98	100	98
	1	8	1	2	2	2	0	2
	2	5	0	0	0	0	0	0
**Proctitis**	0	95	100	100	100	99	99	100
	1	4	0	0	0	1	1	0
	2	1	0	0	0	0	0	0
**Rectal bleeding**	0	92	96	96	93	94	94	91
	1	5	3	2	4	3	3	7
	2	3	1	1	2	2	2	0
	3	0	0	1	1	1	1	2
**Hematuria**	0	99	97	98	98	97	96	95
	1	1	3	2	2	3	4	4
	2	0	0	0	0	0	0	1
**Dysuria**	0	79	89	95	93	98	97	96
	1	20	11	5	7	2	3	4
	2	1	0	0	0	0	0	0
**Urinary frequency**	0	79	94	93	95	93	96	96
	1	18	5	6	5	6	4	4
	2	3	1	1	0	1	0	0
**Retention**	0	89	97	95	96	97	96	96
	1	10	2	3	1	1	2	0
	2	1	1	0	1	0	1	2
	3	0	0	2	2	2	1	2
**Incontinence**	0	87	80	85	90	90	87	87
	1	8	18	14	9	9	10	11
	2	5	2	1	1	1	3	2

### Sexual function

At baseline, 46.6% was always (21.9%) or almost always (24.8%) able to have an erection, while at the first follow-up, only the 10.5% of patients was always (1.9%) or almost always (8.6%) able to have an erection. After 1 year from the end of HT there was a partial recovery of sexual function (17.1% always or almost always) (Table 4).

**Table 4:** Sexual function at baseline, 3 months after RT and 1 year after HT

**Response**	Sexual function*					
	**At baseline**		**3 months after RT**		**1 year after HT**	
	**n.**	**%**	**n.**	**%**	**n.**	**%**
**Always**	23	21.9	2	1.9	10	9.6
**Almost always**	26	24.8	9	8.6	8	7.6
**Sometimes**	21	20	15	14.3	20	19
**Almost never**	12	11.4	24	22.9	25	23.8
**Never**	11	10.5	43	40.9	21	20
**Not applicable or not answer**	12	11.4	12	11.4	21	20

*Responses were to the question, “When sexually excited, are you able to get an erection?”

## Discussion

Hypofractionated radiotherapy represents an attractive therapeutic option for tumors with low proliferation grade such as prostate cancer. The difference in α/β ratio between the tumor itself and surrounding normal tissues may lead to a therapeutic gain as well as an advantage for the patient and the health care system because the reduced overall duration of the treatment.

In the last decade, several randomized trial compared HyRT vs. conventional RT showing good toxicity rates and local control but only in a few studies was an adequate dose delivered [4,5,7,10].

In particular, Lukka et al. delivered 66 Gy in 33 fractions (conventional RT) vs. 52.5 Gy in 20 fractions over 28 days (HyRT), while Yeoh et al. compared hypofractionated radiotherapy (55 Gy in 20 fractions within 4 weeks) vs. a conventionally fractionated RT (64 Gy in 32 fractions within 6.5 weeks) [4,5].

Dearnaley et al. compared a conventional schedule of 74 Gy in 37 fractions with two hypofractionated high-dose intensity-modulated schedules with a total dose 60 Gy or 57 Gy, delivered in 20 or 19 fractions of 3 Gy per fraction, respectively [10]. All patients were also submitted to receive ADT for 4–6 months. The cumulative rates of late GI and GU toxicities ≥ Grade 2 showed not statistically significant differences between the three groups while survival analysis was not reported.

Arcangeli et al. randomized 168 high-risk prostate cancer patients to receive HyRT (62 Gy in 20 fractions, 4 fractions/week) or conventionally fractionated (80 Gy in 40 fractions) three-dimensional conformal radiotherapy to the prostate and seminal vesicles [7]. All patients were also submitted to 9 months-ADT started 2 months before RT. The reported ≥ Grade 2 GI and GU late toxicities were similar in both groups, while the 4-year actuarial freedom from biochemical failure was significantly different for patients treated with HyRT vs. conventional fractionation (82% vs. 60%, respectively; p = 0.004).

The use of HyRT in prostate cancer patients has also been investigated in several recent phase II studies that demonstrated good biochemical control rates depending on risk stratification and acceptable toxicities (≥ Grade 2 GU and GI late toxicities ranging from 3% to 25%) [8,9,11-13,22]. Therefore, further randomized studies are needed to confirm these data. In the present study, 5.7% patients experienced ≥ Grade 2 GI toxicity and 9.5% patients had ≥ Grade 2 GU toxicity. Our results are comparable to those reported in the literature.

Zelefsky et al. found that high-dose IGRT is associated with a low rate of late urinary toxicity and with an improvement in biochemical tumor control among high-risk patients [23]. Differently, in our study the use of IGRT was associated to a decreased late GI toxicity and combined late GI plus GU toxicities. Such different results obtained by Zelefsky et al. can be explained by the use of IMRT instead of 3D-CRT, a technique able to spare the bladder neck that is responsible of late GU toxicities after radiotherapy. On the contrary, the reduction in late GI toxicities we observed are possibly due to several factors such as: the role of MRI during the planning that reduced the CTV, the reduced PTV margin, and the daily CBCT that decreased set-up errors. Acute toxicity rates were similar in both groups, even if a trend of significance for better outcome was found regarding the incidence of acute GI toxicity in favor of IGRT.

The association of ADT to HyRT is still an open question. A preliminary report of the French trial GETUG 14 regarding patients affected by intermediate-risk cancer treated with dose escalated RT vs. dose escalated RT plus short course ADT showed a statistically significant increased 3-year biochemical progression-free survival; no difference was found between groups in terms of combined biochemical and local tumor control [24]. A retrospective study by Zumsteg et al. demonstrated that short-term ADT improves prostate specific antigen recurrence-free survival, distant metastasis and prostate cancer specific mortality in patients with intermediate-risk cancer undergoing dose-escalated external beam radiation therapy [25]. Despite the fact that the short course ADT is useful for dose escalated RT in intermediate risk patients, the association with HyRT is still controversial. A recent report by Faria et al. showed a 5-year actuarial biochemical recurrence free survival of 95.4% in intermediate-risk prostate cancer patients treated with HyRT alone (total dose of 66 Gy in 22 fractions) after a median follow-up of 51 months [9]. The authors explained these results with a short overall treatment time (30.5 days) and the high biologically equivalent dose delivered. Similarly to Arcangeli et al. in our study all patients underwent 9 months ADT reaching a 3-years bNED of 93.7%. The results achieved by Faria et al. are similar to those obtained in our study suggesting that HT may be avoided when HyRT is delivered since HT don’t seems to improve the results of HyRT in intermediate-risk prostate cancer patients. Furthermore, Kupelian et al. found that the use of hormonal therapy before or after HyRT (70 Gy at 2.5 Gy per fraction) was an independent predictor of a worse outcome [13]. However, to determine the exact role of HT in the era of HyRT in intermediate-risk prostate cancer randomized trials are needed.

The strength of this paper is that a novel HyRT schedule was examined in 105 patients affected by intermediate-risk prostate cancer. The majority of studies on prostate cancer includes different categories of risk making the results not generalizable. Indeed, it should be taken into account that intermediate-risk prostate cancer itself would represent a clinically non-uniform group of disease characterized by a wide range of clinical behavior [26].

On the other hand, there are several limits of this study including the low median follow-up and the different RT technique used. Moreover, the study was unpowered to detect differences in terms of acute toxicities between the two groups of patients, probably due to the small number of patients treated without IGRT. Despite these limits we found a clearly advantage of using daily IGRT in HyRT in terms of reduced ≥ Grade 2 late toxicities.

## Conclusions

In conclusion, the HyRT schedule represents a well-tolerated treatment with acceptable rates of both acute and late toxicity, able to achieve a high bNED in intermediate-risk prostate cancer; but a longer follow-up is needed. The use of daily IGRT is beneficial for reducing the incidence of late toxicities but further studies are required to confirm these results.

## Competing interest

The authors declare that they have no competing interests.

## Authors’ contributions

MV, SB and MFO designed the study; SB, LA and FT collected and analyzed data; MV, SB, LA wrote the manuscript; MV, MFO, VDS and RME critically reviewed/revised the article. All authors read and approved the final manuscript.

## References

[B1] Viani GA Stefano EJ Afonso SL Higher than conventional radiation doses in localized prostate cancer treatment: a meta-analysis of randomized controlled trials Int J Radiat Oncol Biol Phys 2009 74 1405 1418 10.1016/j.ijrobp.2008.10.091 19616743

[B2] Fowler JF The radiobiology of prostate cancer including new aspects of fractionated radiotherapy Acta Oncol 2005 44 265 276 10.1080/02841860410002824 16076699

[B3] Fowler JF Ritter MA Chappell RJ What hypofractionated protocols should be tested for prostate cancer? Int J Radiat Oncol Biol Phys 2003 56 1093 1104 10.1016/S0360-3016(03)00132-9 12829147

[B4] Lukka H Hayter C Julian JA Randomized trial comparing two fractionation schedules for patients with localized prostate cancer J Clin Oncol 2005 23 6132 6138 10.1200/JCO.2005.06.153 16135479

[B5] Yeoh EE Botten RJ Butters J Hypofractionated versus conventionally fractionated radiotherapy for prostate carcinoma: final results of phase III randomized trial Int J Radiat Oncol Biol Phys 2011 81 1271 1278 10.1016/j.ijrobp.2010.07.1984 20934277

[B6] Michalski J Winter K Roach M Clinical outcome of patients treated with 3D conformal radiation therapy (3D-CRT) for prostate cancer on RTOG 9406 Int J Radiat Oncol Biol Phys 2012 83 e363 e370 10.1016/j.ijrobp.2011.12.070 22633552 PMC3361689

[B7] Arcangeli G Saracino B Gomellini S A prospective phase III randomized trial of hypofractionation versus conventional fractionation in patients with high-risk prostate cancer Int J Radiat Oncol Biol Phys 2010 78 11 18 10.1016/j.ijrobp.2009.07.1691 20047800

[B8] Yassa M Fortin B Fortin MA Combined hypofractionated radiation and hormone therapy for the treatment of intermediate-risk prostate cancer Int J Radiat Oncol Biol Phys 2008 71 58 63 10.1016/j.ijrobp.2007.09.004 18406883

[B9] Faria S Dal Pra A Cury F Treating intermediate-risk prostate cancer with hypofractionated external beam radiotherapy alone Radiother Oncol 2011 101 486 489 10.1016/j.radonc.2011.07.005 21864925

[B10] Dearnaley D Syndikus I Sumo G Conventional versus hypofractionated high-dose intensity-modulated radiotherapy for prostate cancer: preliminary results from the CHHiP randomised controlled trial Lancet Oncol 2012 13 43 54 10.1016/S1470-2045(11)70293-5 22169269

[B11] Zilli T Jorcano S Rouzaud M Twice-weekly hypofractionated intensity-modulated radiotherapy for localized prostate cancer with low-risk nodal involvement: toxicity and outcome from a dose escalation pilot study Int J Radiat Oncol Biol Phys 2011 81 382 389 10.1016/j.ijrobp.2010.05.057 20884129

[B12] Martin JM Rosewall T Bayley A Phase II trial of hypofractionated image-guided intensity-modulated radiotherapy for localized prostate adenocarcinoma Int J Radiat Oncol Biol Phys 2007 69 1084 1089 10.1016/j.ijrobp.2007.04.049 17606331

[B13] Kupelian PA Willoughby TR Reddy CA Hypofractionated intensity-modulated radiotherapy (70 Gy at 2.5 Gy per fraction) for localized prostate cancer: Cleveland Clinic experience Int J Radiat Oncol Biol Phys 2007 68 1424 1430 10.1016/j.ijrobp.2007.01.067 17544601

[B14] Madsen BL Hsi RA Pham HT Stereotactic hypofractionated accurate radiotherapy of the prostate (SHARP), 33.5 Gy in five fractions for localized disease: first clinical trial results Int J Radiat Oncol Biol Phys 2007 67 1099 1105 10.1016/j.ijrobp.2006.10.050 17336216

[B15] Ju AW Wang H Oermann EK Hypofractionated stereotactic body radiation therapy as monotherapy for intermediate-risk prostate cancer Radiat Oncol 2013 8 30 10.1186/1748-717X-8-30 23369294 PMC3570380

[B16] Valeriani M Monaco F Osti MF Hypofractionated radiotherapy with or without IGRT in prostate cancer: preliminary report of acute toxicity Anticancer Res 2011 31 3555 3558 21965777

[B17] Mohler J Bahnson RR Boston B NCCN clinical practice guidelines in oncology: Prostate cancer J Natl Compr Canc Netw 2010 8 162e20 20141676 10.6004/jnccn.2010.0012

[B18] Heemsbergen WD Peeters STH Koper PCM Acute and late gastrointestinal toxicity after radiotherapy in prostate cancer patients: consequential late damage Int J Radiat Oncol Biol Phys 2006 66 3 10 10.1016/j.ijrobp.2006.03.055 16814954

[B19] Cox J Stetz J Pajak T Toxicity criteria of the Radiation Therapy Oncology Group (RTOG) and the European Organization for the Treatment and Research of Cancer (EORTC) Int J Radiat Oncol Biol Phys 1995 31 1341 1346 10.1016/0360-3016(95)00060-C 7713792

[B20] Waterhouse J Metcalfe MC Development of the sexual adjustment questionnaire Oncol Nurs Forum 1986 13 53 59 3635138

[B21] Roach M 3rd Hanks G Thames H Jr Defining biochemical failure following radiotherapy with or without hormonal therapy in men with clinically localized prostate cancer: recommendations of the RTOG-ASTRO Phoenix Consensus Conference Int J Radiat Oncol Biol Phys 2006 65 965 974 10.1016/j.ijrobp.2006.04.029 16798415

[B22] Wu J Brasher P El-Gayed A Phase II study of hypofractionated image-guided radiotherapy for localized prostate cancer: outcomes of 55 Gy in 16 fractions at 3.4 Gy per fraction Radiother Oncol 2012 103 210 216 10.1016/j.radonc.2011.12.020 22280805

[B23] Zelefsky MJ Kollmeier M Cox B Improved clinical outcomes with high-dose image guided radiotherapy compared with Non-IGRT for the treatment of clinically localized prostate cancer Int J Radiation Oncol Biol Phys 2012 84 125 129 10.1016/j.ijrobp.2011.11.047 22330997

[B24] Dubray BM Beckendorf V Guerif S Does short-term androgen depletion add to high-dose radiotherapy (80 Gy) in localized intermediate-risk prostate cancer? Intermediate analysis of GETUG 14 randomized trial (EU-20503/NCT00104741) [abstract] Proc Am Soc Clin Oncol 2011 29 s4521

[B25] Zumsteg ZS Spratt DE Pei X Short-term androgen-deprivation therapy improves prostate cancer-specific mortality in intermediate-risk prostate cancer patients undergoing dose-escalated external beam radiation therapy Int J Radiation Oncol Biol Phys 2012 in press 10.1016/j.ijrobp.2012.07.2374 22981709

[B26] Zumsteg ZS Zelefsky MJ Short-term androgen deprivation therapy for patients with intermediate-risk prostate cancer undergoing dose-escalated radiotherapy: the standard of care? Lancet Oncol 2012 13 e259 e269 10.1016/S1470-2045(12)70084-0 22652234

